# Ionizing Radiation Induces Endothelial Inflammation and Apoptosis via p90RSK-Mediated ERK5 S496 Phosphorylation

**DOI:** 10.3389/fcvm.2018.00023

**Published:** 2018-03-14

**Authors:** Hang Thi Vu, Sivareddy Kotla, Kyung Ae Ko, Yuka Fujii, Yunting Tao, Jan Medina, Tamlyn Thomas, Megumi Hada, Anil K. Sood, Pankaj Kumar Singh, Sarah A. Milgrom, Sunil Krishnan, Keigi Fujiwara, Nhat-Tu Le, Jun-Ichi Abe

**Affiliations:** ^1^Department of Cardiology, The University of Texas MD Anderson Cancer Center, Houston, TX, United States; ^2^Department of Cardiovascular Sciences, Houston Methodist Research Institute, Houston, TX, United States; ^3^Texas A&M Chancellor Research Initiative, Prairie View A&M University, Prairie View, TX, United States; ^4^Department of Gynecologic Oncology and Reproductive Medicine, The University of Texas MD Anderson Cancer Center, Houston, TX, United States; ^5^Department of Radiology Oncology, The University of Texas MD Anderson Cancer Center, Houston, TX, United States

**Keywords:** Ionizing radiation, p90RSK, ERK5, EC inflammation, EC apoptosis

## Abstract

Adverse cardiovascular events are a leading nonmalignant cause of morbidity and mortality among cancer survivors who have been exposed to ionizing radiation (IR), but the exact mechanism of the cardiovascular complications induced by IR remains unclear. In this study we investigated the potential role of the p90RSK-ERK5 module in regulating IR-induced endothelial cell inflammation and apoptosis.

Whole body radiation of mice with 2 Gy γ-ray significantly increased endothelial VCAM-1 expression; especially in the disturbed flow area *in vivo*. *In vitro* studies showed that IR increased p90RSK activation as well as subsequent ERK5 S496 phosphorylation in cultured human endothelial cells (ECs). A specific p90RSK inhibitor, FMK-MEA, significantly inhibited both p90RSK activation and ERK5 S496 phosphorylation, but it had no effect on IR-induced ERK5 TEY motif phosphorylation, suggesting that p90RSK regulates ERK5 transcriptional activity, but not its kinase activity. In fact, we found that IR-induced NF-kB activation and VCAM-1 expression in ECs were significantly inhibited by the over-expression of S496 phosphorylation site mutant of ERK5 (ERK5 S496A) compared to overexpression of wild type ERK5. Furthermore, when ECs were exposed to IR, the number of annexin V positive cells increased, and overexpression of ERK5 S496A, but not wild type ERK5, significantly inhibited this increase.

Our results demonstrate that IR augmented disturbed flow-induced VCAM-1 expression *in vivo*. Endothelial p90RSK was robustly activated by IR and subsequently up-regulated ERK5 S496 phosphorylation, inflammation, and apoptosis in ECs. The EC p90RSK-ERK5 signaling axis can be a good target to prevent cardiovascular events after radiation therapy in cancer patients.

## Introduction

Radiation therapy (RT) is a critical component of the management of many malignancies, including primary thoracic malignancies, breast cancer, and lymphoma, but it is now known that RT causes various cardiovascular (CV) disorders such as coronary artery disease, congestive heart failure, and valvular disorders, resulting in morbidity and mortality among cancer survivors. For example, in survivors of Hodgkin lymphoma, RT to the chest area increases the risk of CV disease by up to 7-fold based on multivariate analyses controlling for other risk factors (1), and heart disease is the leading cause of non-oncologic death (2). Likewise, RT has been associated with significantly increased mortality due to CV disease in breast cancer survivors (3–5). In patients with locally advanced non-small cell lung cancer, the 2 year incidence of grade ≥3 CV toxicity after RT is 11% according to a combined analysis of 4 prospective studies (6), and deaths related to excess heart exposure may offset any benefit of increasing the radiation dose to control the disease (7). Thus, the cardiotoxic effects of RT are a serious concern. The cardiovascular effects of RT may be amplified in patients who also receive cardiotoxic chemotherapeutic agents, such as anthracyclines and trastuzumab. Although the role of IR in EC inflammation has been reported (8, 9), the exact molecular mechanisms remain unclear.

Shear stress is imposed directly on the luminal surface of blood vessels covered by a thin monolayer of ECs. There are two types of flow, which can differentially affect EC structure and function by regulating different cellular mechanosignaling pathways. Each of the mechanosignaling mechanisms will then activate shear stress response promoter elements and transcription factors in ECs residing in areas exposed to the two different types of flow and determine the cellular phenotype (10–13). For example, atherosclerosis (AS) lesions are rare in areas exposed to laminar flow. It is known that steady laminar flow (s-flow:10–20 dyne/cm^2^) induces anti-inflammatory signaling and anti-atherogenic gene expression in cultured ECs (14, 15). S-flow suppresses the expression of inflammatory chemokines and adhesion molecules (VCAM-1, ICAM-1, and E-selectin) while maintaining the production of athero-protective factors such as nitric oxide (NO) and EC nitric oxide synthase (eNOS) (16–19). ERK5, a member of the mitogen-activated protein kinase family, is unique in that it is not only a kinase but also a transcriptional co-activator with a unique C-terminus transactivation domain (20, 21). It has been suggested that Krüppel-like factor 2 (KLF2) plays a crucial role in s-flow-induced anti-inflammatory effects via ERK5 activation (20, 22). Atherosclerotic plaques localize to areas of disturbed flow (d-flow) found in regions where vessels curve acutely, bifurcate, or branch. D-flow is pro-atherogenic; induces inflammation, apoptosis, and proliferation of ECs and reduces vascular reactivity. p90RSK is a unique serine/threonine kinase with two distinct functional kinase domains (23) that has been well-characterized by our group for its role in heart failure (24, 25). Our group has reported that d-flow induces phosphorylation of p90RSK, which then allows p90RSK to bind the C-terminus region of ERK5 (amino acids 571–807). This binding enables p90RSK to phosphorylate ERK5 at S496 (26) and inhibits the transcriptional activity of ERK5 (26). This inhibition ablates the promotor activity of KLF2 and eNOS activity. These series of molecular events lead to EC inflammation and dysfunction.

In this study, we found that IR enhanced EC inflammation, especially in the disturbed flow area compared to the laminar flow area *in vivo*. We also found that IR increased p90RSK activation and subsequent ERK5 S496 phosphorylation. Overexpression of an ERK5 S496A mutant, but not wild type ERK5, in ECs significantly inhibited the expression of NF-κB and VCAM-1 and apoptosis. To our knowledge, this is the first report that shows the crucial role of p90RSK-mediated ERK5 S496 phosphorylation in regulating IR-induced EC inflammation and apoptosis. The endothelial p90RSK-ERK5 axis can be a good target to prevent cardiovascular events after radiation therapy in cancer patients. In addition, since endothelial inflammation is a hallmark of radio-resistance, IR-induced activation of the endothelial p90RSK-ERK5 signaling cascade may contribute to radiation sensitivity of cancers.

## Methods

### Antibodies and Reagents

Antibodies were purchased from the following vendors: anti-p90RSK1 (C-21, #SC-231) and anti-VCAM-1 (SC-8304) from Santa Cruz Biotechnology (Dallas, TX); anti-phospho-p90RSK (Ser380, #9341), anti-phospho-ERK5 (Thr218/Tyr220, #3371L), and anti-ERK5 (#3372) from Cell Signaling (Danvers, MA); anti-tubulin (T-5168) from Sigma (St. Louis, MO); anti-VE-cadherin (#555289) from BD Transduction Laboratory (San Diego, CA); and anti-phopho-specific ERK5 S496 antibody (#A02812) from Boster Bio (Pleasanton, CA).

### Generation of Plasmids

Plasmids containing wild type (WT) p90RSK (WT-p90RSK) and dominant negative (kinase dead) p90RSK (DN-p90RSK) were generated as previously described (26). Gal4-ERK5 was created by inserting mouse ERK5 isolated from pcDNA3.1-ERK5 into the BamH1 and Not1 sites of the pBIND vector (Invitrogen, Carlsbad, CA). An adenovirus vector containing constitutively active (CA)-MEK5α was subcloned into the pENTR vector (Invitrogen) using specific enzyme sites, and then a recombinase reaction was performed to get a pDEST-based vector following manufacture’s instruction (#K4930-00, ViraPower Adenoviral Expression System, Promega, Madison, WI). All constructs were verified by DNA sequencing by using vector specific primers.

#### Irradiation

Cells and mice were irradiated on the Cesium-137 Research-Irradiator “Mark-I Model M68A” (J. L. Shepherd and Associates, San Fernando, CA). The unit consists of sealed pencil-like (37 cm long) Radio-Active Source Cesium-137 producing 662 keV Gamma radiation. The Petri dishes were placed on an Acrylic stand, 10.8 cm above the upper floor-plate, to ensure dose-uniformity within 5% (95% iso-dose circle). C57BL/6 mice were placed in the cylindrical container (inner Diameter 20.2 cm, height 13.4 cm). Thin black plastic-strips were arranged to create wedge-shaped partitions for 8 mice. A round top-cover of clear plastic with a wedged-cut was used to place and retrieve mice one at a time. The mice-loaded container was placed on an Acrylic stand 30.1 cm above the axis of pencil-like Cesium-Source. A dose of 2 Gy was delivered in this geometry. The mice container diameter (20.2 cm) ensured dose-uniformity within 5% (the 95% iso-dose circle has a diameter of 24 cm). In-air dose-rate at the irradiation height was measured with an ion-chamber employing the Task-Group Report 61 of the AAPM (American Association of Physicists in Medicine). The chamber had been calibrated by the M. D. Anderson’s ADCL (Acredited Dosimetry Calibration-Laboratory). The chamber-calibration is traceable to NIST (National Institute of Standards and Technology). Experimental procedures were approved by the Institutional Care and Use Committee (IACUC) at Texas A&M Institute of Biosciences and Technology and the University of Texas MD Anderson Cancer Center.

### En Face Staining of Mouse Aortas

C57Bl/6 male mice were used for this study. The disturbed flow (d-flow) and undisturbed laminar blood flow (l-flow) areas in the mouse aorta were identified based on generally accepted anatomical locations where such flow patterns are known to occur (27–29). We performed *en face* staining and confocal imaging as described in our previous reports (26, 28). Experimental procedures were approved by the Institutional Care and Use Committee (IACUC) at Texas A&M Institute of Biosciences and Technology and the University of Texas MD Anderson Cancer Center.

### Transient Over-Expressions

For protein overexpression, ECs were transfected with Flag-tagged ERK5 WT, and ERK5-S496A mutant using GIBCO Opti-MEM reduced serum medium (cat. no. 31985070; Thermo Fisher Scientific, Waltham, MA) containing Plus and Lipofectamine reagents (cat. no. 11514015 and 18324020, respectively; Life Technologies, Carlsbad, CA) as we described previously (26).

### PathDetect in Vivo Signal Transduction Pathway Reporting System

NF-κB activity was assayed using the PathDetect Signal Transduction Pathway trans-Reporting Systems (Stratagene, San Diego, CA) as we described previously (30).

### Western Blotting

We applied an equal amount of proteins from control and treatment samples into each well of SDS-PAGE gels and performed Western blotting using specific antibodies for the indicated molecules in the figure as we described previously (31). Nitrocellulose membranes were incubated with primary antibodies diluted 500–1,000 times overnight at 4°C. After blocking with 3% BSA for one hour, the membranes were incubated with HRP-conjugated appropriate (goat anti-mouse or anti-rabbit) secondary antibodies diluted 4,000–5,000 times. Blots were developed using an ECL reagent, imaged using Film Processor SRX-101A (Konika Minolta Medical Graphics, Tokyo, Japan), and signal intensities quantified by densitometry using ImageJ software as we described (26).

### Cell Culture and Transfection

We purchased primary human aortic endothelial cells (HAECs) from Invitrogen (Cat no-C0065C) and were grown in complete Endothelial Cell Medium (ECM, Sciencell Research Laboratories, Carlsbad, CA) supplemented with endothelial growth factors along with 5% FBS and 1% penicillin/streptomycin. Human umbilical vein ECs (HUVECs) were obtained from collagenase digested umbilical cord veins (32) and collected in M200 medium supplemented with LSGS (Cascade Biologics Inc., Portland, OR) and 2% fetal bovine serum (FBS) (Atlanta Biologicals Inc., Lawrenceville, GA) as we described previously (26). HUVECs were cultured on 0.2% gelatin pre-coated dishes. All the cells were maintained at 37°C in a humidified atmosphere of 5% CO2. For transient expression experiments, 70 to 80% confluent cells were transfected with cDNA using Opti-MEM containing Plus-Lipofectamine as we previously described (33). After 4 h of transfection, Opti-MEM was replaced with the complete M200 medium.

### EC Apoptosis and VCAM-1 Expression

After transfection with flag-tagged ERK5 wild type or the S496A mutant, ECs were exposed to d-flow (24 h) and harvested by incubating with EDTA (10 mM in PBS). Cells were stained with annexin V-fluorescein isothiocyanate (Annexin V-FITC Apoptosis Detection Kit, cat. no. ab14085, Abcam, Cambridge, MA) according to manufacturer’s instructions. Annexin V-positive cells were quantified using a BD Accuri C6 Flow Cytometer and the FlowJo software program. For detecting VCAM-1 expression, each sample was divided into 2 parts: one part was probed with an Alexa fluor **®** 488**-**conjugated mouse monoclonal antibody against human VCAM-1 (RD systems FAB5649G) while the other was probed with Alexa fluor **®** 488**-**conjugated isotype-matched control mouse IgG2a (RD systems: #IC003G). Both were subjected a BD Accuri C6 Flow Cytometer and assayed according to manufacturer’s instructions.

### Statistics

Differences between two independent groups were determined using the Student *t*-test (two-tailed) and, when applicable, one-way ANOVA followed by Bonferroni post hoc testing for multiple group comparisons using GraphPad Prism (GraphPad Software, SanDiego) was employed. When groups exhibited unequal variances, Welch’s ANOVA was used to perform multiple group comparisons. *P* values less than 0.05 were considered statistically significant and are indicated by asterisks in the figures. *P* values <0.01 are indicated by two asterisks.

## Results

### IR Induced EC Inflammation, Especially in Disturbed Flow Area *in Vivo*

**Figure 1:**
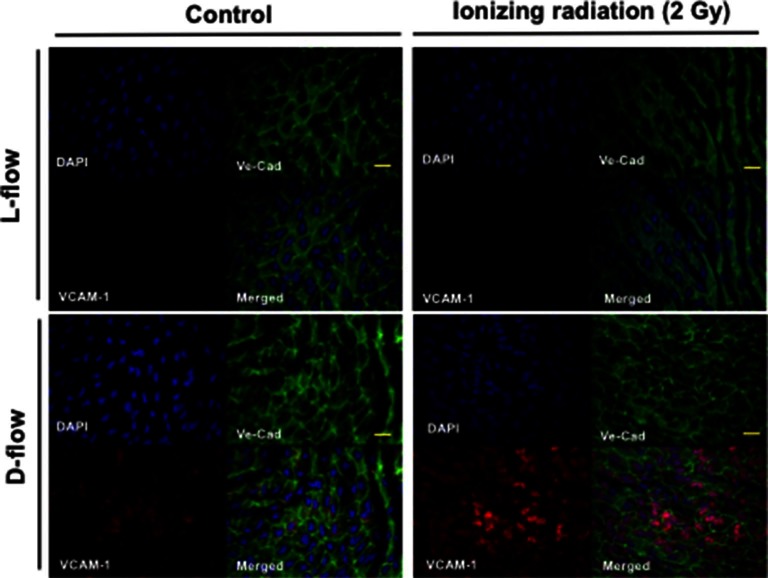
IR enhanced d-flow-induced VCAM-1 expression. En face preparations of the aortic arch after 19 h of IR 0 (control) and 2 Gy-treated mice were tripple-stained with anti-Ve-cadherin (Ve-Cad as an EC marker), DAPI, and anti–VCAM-1. Representative confocal images from three independent experiments are shown. Scale: 20 µm

Mancuso et al. have reported that atherosclerotic lesion formation in irradiated athero-prone ApoE^−/−^ mice was accelerated, especially in the d-flow area (34). Therefore, to determine the role of IR in EC inflammation *in vivo*, aortas from male C57BL/6 mice exposed to IR (0–2 Gy) were isolated 19 h after IR, and *en face* preparations were stained with anti-VCAM-1. 2 Gy of whole-body radiation did not have any significant detrimental life threating effects on mice. As we have reported previously, we found that VCAM-1 expression was higher in the d-flow area compared with the laminar flow (l-flow) area. Interestingly, we found that IR treatment significantly enhanced VCAM-1 expression only in the area exposed to d-flow, and we could not find any significant increase in VCAM-1 expression after IR treatment in the l-flow area (Figure 1). These data suggest the interplay between d-flow and IR in EC inflammation and support the idea that IR initially affects EC inflammation at the athero-prone area.

### IR Increased ERK5 S496 Phosphorylation via p90RSK Activation

**Figure 2:**
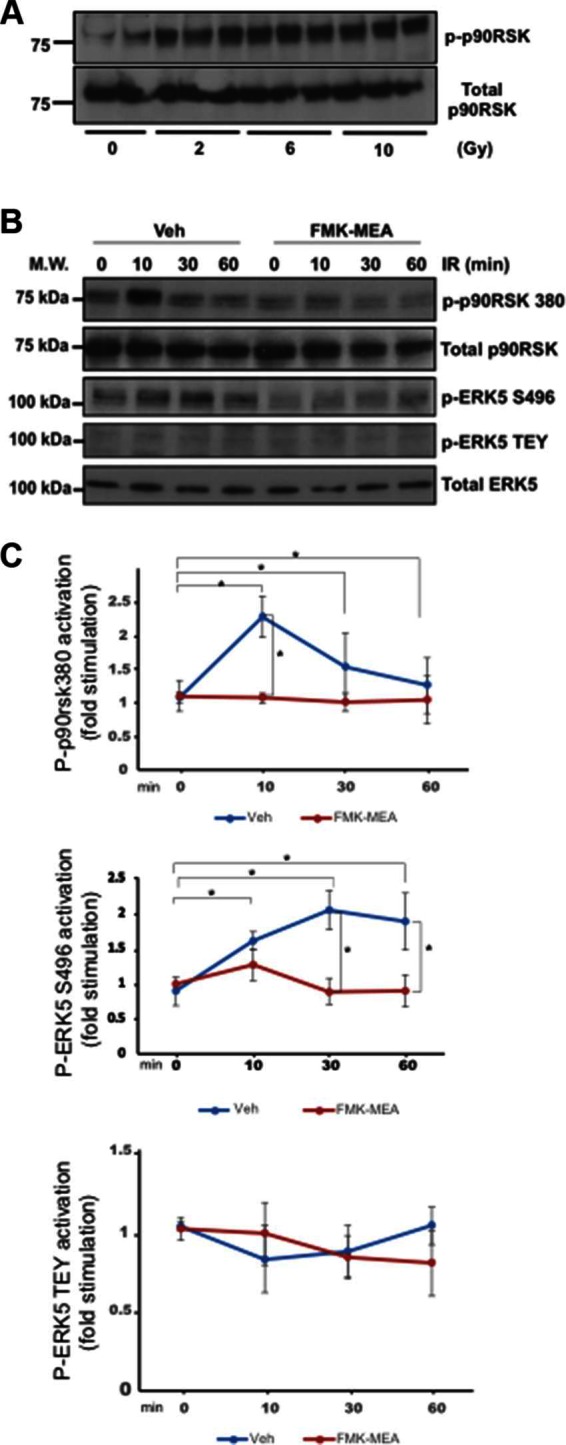
IR-induced p90RSK activation lead to ERK5 S496 phosphorylation, but not ERK5 TEY motif phosphorylation. **(A)** HUVECs were treated with IR by the indicated doses, and 30 min after IR, ECs were collected and p90RSK activity was detected by Western blotting with anti–p-p90RSK and anti-p90RSK antibodies. Representative images from three independent experiments are shown. **(B)** HAECs were pre-treated with FMK-MEA (5 µM) for 30 min and treated by IR (2 Gy) for the indicated times. ECs were collected, and total p90RSK, p90RSK phosphorylation, ERK5 S496 phosphorylation, ERK5 TEY motif phosphorylation, and total ERK5 were detected by Western blotting with anti-p90RSK, anti-phospho-p90RSK (S380), anti-phospho-ERK5 (S496), anti-phospho-ERK5 (TEY motif), and anti-ERK5. Representative images from three independent experiments are shown. **(C)** Quantification of IR-induced p90RSK activation (S380 phosphorylation; top), ERK5 S496 phosphorylation (middle), and ERK5 activation (TEY motif phosphorylation; bottom) is shown after normalization by total protein levels. Data represent mean ± SEM (*n* = 3).

Previously, we have reported the key role of p90RSK activation in regulating ERK5 S496 phosphorylation (26). We found that p90RSK activation was dose-dependently increased by IR (Figure 2A). In addition, IR (2Gy) significantly increased phosphorylation of ERK5 S496, but we found no change in ERK5 TEY motif phosphorylation after IR (Figure 2B,C), which is directly related to ERK5 kinase activation. In addition, we also found that the pre-treatment of cells with a p90RSK specific inhibitor, FMK-MEA, significantly inhibited ERK5 S496 phosphorylation, but not TEY motif phosphorylation (Figure 2B,C). These data support the critical role of p90RSK activation in regulating IR-induced ERK5 S496 phosphorylation.

### EC Inflammation Was Regulated by ERK5 S496 Phosphorylation

**Figure 3:**
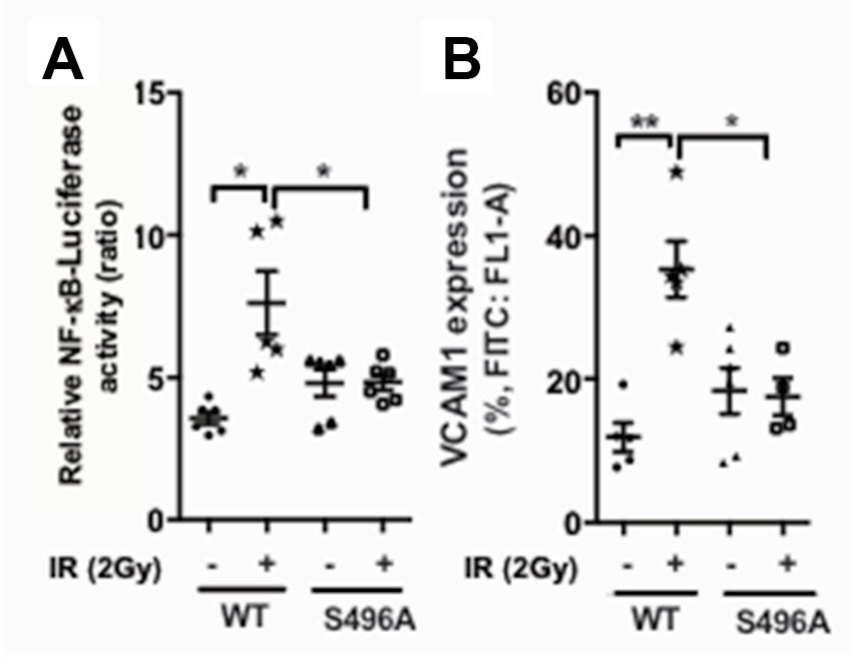
ERK5 S496 phosphorylation is critical for IR-induced EC inflammation. HUVECs were co-transfected with the NF-κB-Luc luciferase reporter vector and the pRL-CMV vector (renilla luciferase activity, internal control) together with the expression vector encoding the flag-tagged ERK5 wild type (WT) or the S496A mutant. Transfected cells were then exposed to gamma radiation (2 Gy) and harvested after 24 h. **(A)** NF-κB activation was quantified by measuring relative luciferase activity using a dual-luciferase reporter system. Shown are relative luciferase activity, presented as firefly luciferase/renilla luciferase activity ratio. (*n* = 6) **(B)** VCAM1 expression was determined by measuring percentage of positive VCAM1-staining cells using the BD Accuri C6 Flow Cytometer system using the FL1 533/30 nm filter. (*n* = 5)

To determine the role of ERK5 S496 phosphorylation in EC inflammation, we overexpressed either wild type ERK5 or the ERK5 S496A mutant in ECs and examined whether IR can increase NF-kB activation and VCAM-1 expression (Figure 3). We found that IR significantly increased both NF-kB and VCAM-1 expression in cells overexpressing wild type ERK5 but that these increases were not observed in cells overexpressing the ERK5 S496A mutant.

### IR-Induced EC Apoptosis Was Inhibited by Overexpression of ERK5 S496A Mutant

**Figure 4:**
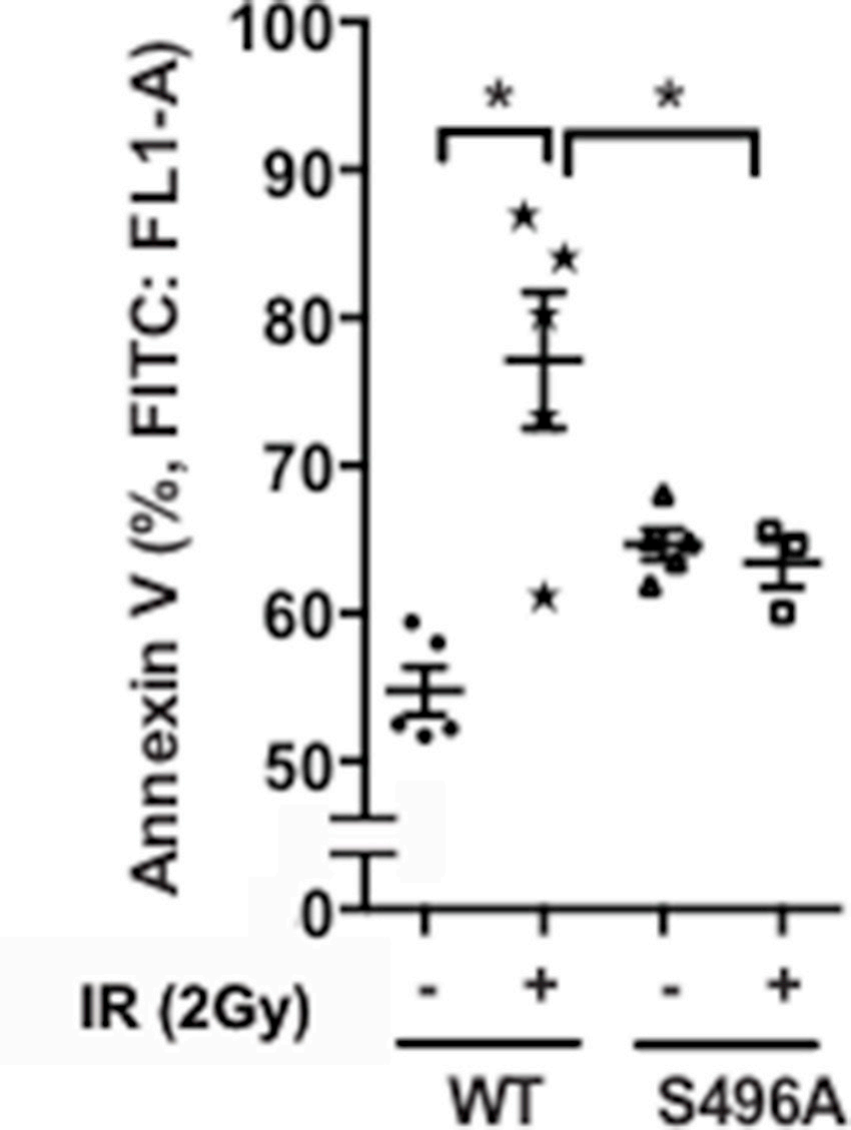
ERK5 S496 phosphorylation is critical for IR-induced apoptosis. HUVECs were transfected with the expression vector encoding the flag-tagged ERK5 wild type (WT) or the S496A mutant. Transfected cells were then exposed to gamma radiation (2 Gy), and harvested after 60 h. Cell apoptosis was determined by measuring percentage of Annexin V- positive cells (Abcam’s Annexin V-FITC Apoptosis Detection Kit) using the BD Accuri C6 Flow Cytometer system with the FL1 533/30 nm filter. Shown are mean ± SEM, *n* = 3–6.

The anti-apoptotic role of ERK5 has been reported (33). Therefore, we examined the role of ERK5 S496 phosphorylation in IR-induced EC apoptosis. A significant increase of annexin V expression after IR was observed, and overexpression of ERK5 S496A mutant significantly inhibited IR-induced apoptosis (Figure 4). These results are consistent with the crucial role played by ERK5 S496 phosphorylation in IR-induced EC apoptosis. All the data obtained in this study indicate that IR causes ERK5 S496 phosphorylation and that this phosphorylation plays a major role in inducing EC inflammation and apoptosis.

## Discussion

In this study, we found the crucial role of p90RSK-mediated ERK5 S496 phosphorylation in IR-induced EC inflammation and apoptosis. Although it has been well established that IR can induce inflammation, the details of mechanistic insights into this inflammatory response are lacking. We found that p90RSK activity was very sensitive to radiation. This IR-induced p90RSK activation led to increased ERK5 S496 phosphorylation. Previously, we have reported that ERK5 S496 phosphorylation decreased ERK5 transcriptional activity and subsequently inhibited KLF2/4 expression, which can up-regulate NF-kB activation and inflammatory gene expression (26). In the current study first we found that a p90RSK specific inhibitor, FMK-MEA, significantly inhibited IR-induced NF-kB activation and subsequent VCAM-1 expression as well as EC apoptosis. Next, to determine the role of p90RSK-mediated ERK5 S496 phosphorylation in IR-induced EC inflammation and apoptosis, we generated adenovirus containing ERK5 S496A mutant and found that overexpression of ERK5 S496A mutant inhibited not only IR-induced inflammatory responses but also apoptosis in endothelial cells. Taken together, these data support the critical role of p90RSK-mediated ERK5 S496 phosphorylation in EC inflammation and apoptosis. The IR-induced EC apoptosis appears to take place via a new signaling pathway, which is responsible for mediating IR-induced EC dysfunction that leads to atherosclerotic plaque formation.

 As shown in Figure 1, we found that IR increased EC inflammation *in vivo*. Interestingly, we found that the extent of increase of VCAM-1 expression by IR was more evident in the disturbed flow area than in the laminar flow area. Previously, we have reported that disturbed flow, but not laminar flow, increased p90RSK activation (35). Importantly, we also reported that p90RSK expression in the disturbed flow area was higher than that in the laminar flow area (35). Therefore, disturbed flow area is more sensitive to IR, which may explain why VCAM-1 expression is considerably higher in the disturbed flow region than in the laminar flow area. Our studies have raised several new areas of investigation. First, it is important to find out in what way p90RSK is involved in IR-induced VCAM-1 expression. For determining the exact molecular mechanism of IR-induced EC dysfunction *in vivo*, it is crucial to understand what determines the differential IR responses in ECs exposed to two different types of flow and how the signaling pathway(s) activated by IR and those activated by different flow types interact.

The crucial role of reactive oxidative species (ROS) induced by IR in regulating cancer development and the surrounding microenvironment has been reported (36). Since we have reported that p90RSK is a redox-sensitive kinase (37), it is possible that endothelial p90RSK activation is up-regulated by IR-induced ROS. IR can generate ROS via radiolytic hydrolysis and mitochondrial dysfunction (36). Hydrolysis results in decomposition of water by ionizing radiation to hydrogen peroxide, superoxide, hydroxyl radicals and singlet oxygen. These radicals can, in turn, react with organic molecules to generate secondary radicals. These primary and secondary radicals can cause protein oxidation, lipid peroxidation and oxidative DNA damage all of which can be potentially lethal to cells (38). IR can also decrease electron transport chain complex 1 activity and produce ROS persistently (39). It is well known that these ROS production mechanisms can cause EC dysfunction and consequently enhance plaque formation (40, 41). Of note, IR-induced ROS production can generate inflammatory tumor micro-environments and decrease the effectiveness of radiotherapy (36). Therefore, determining the molecular mechanisms of IR-induced EC inflammation is crucial for understanding not only the process of atherosclerosis and cardiovascular events in cancer survivors but also radio-resistance. Further investigation is necessary to clarify these issues.

In this study, we focused on the direct effects of IR on ECs, but it is possible that the contents in tumor cells such as electrolytes and DNA are released after cancer treatment, which then indirectly causes metabolic disturbance and cardiovascular toxicity (42). Not only cancer treatment itself but also the contents of tumor cells after tumor lysis will be important to determine the whole picture of EC activation after IR.

## Ethical Statement

Experimental procedures were approved by the Institutional Care and Use Committee (IACUC) at Texas A&M Institute of Biosciences and Technology and the University of Texas MD Anderson Cancer Center.

## Author Contributions

HV and N-TL performed experiments, interpreted data and wrote the manuscript. SK, KK, YF, YT, JM, TT performed experiments and interpreted data. PS performed radiation study and interpreted data. MH, AKS, SM, SK, KF and J-IA interpreted data, wrote and edited the manuscript.

## Conflict of Interest Statement

The authors declare that the research was conducted in the absence of any commercial or financial relationships that could be construed as a potential conflict of interest.
